# 3-[(5-Methyl­furan-2-yl)methyl­ene]-1,5-dioxaspiro­[5.5]undecane-2,4-dione

**DOI:** 10.1107/S160053680902947X

**Published:** 2009-07-29

**Authors:** Wu-Lan Zeng, Hua-Xiang Zhang, Fang-Fang Jian

**Affiliations:** a Microscale Science Institute, Department of Chemistry and Chemical Engineering, Weifang University, Weifang 261061, People’s Republic of China

## Abstract

There are two crystallographically independent mol­ecules in the asymmetric unit of the title compound, C_15_H_16_O_5_. In each, the 1,3-dioxane ring is in an envelope conformation with the C atom common to the cyclo­hexane ring forming the flap. The dihedral angles between the five essentially planar [maximum deviations from the least-squares planes of 0.049 (3) and 0.042 (3) Å] atoms of the 1,3-dioxane ring and the furan ring in the two mol­ecules are 7.15 (1) and 6.80 (1)°. The crystal structure is stabilized by weak inter­molecular C—H⋯O hydrogen bonds.

## Related literature

For background to the applications of spiro compounds, see: Yaozhong *et al.* (1998 ▶); Lian *et al.* (2008 ▶); Wei *et al.* (2008 ▶). For the crystal structure of 3-(furan-2-ylmethyl­ene)-1,5-dioxa­spiro­[5.5]undecane-2,4-dione, see: Zeng & Jian (2009 ▶).
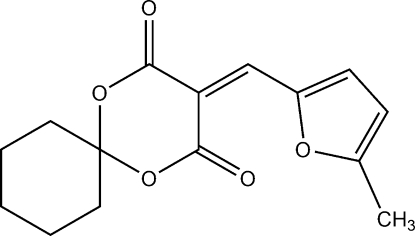


## Experimental

### 

#### Crystal data

C_15_H_16_O_5_
*M* *_r_* = 276.28
Monoclinic, 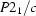

*a* = 19.314 (4) Å
*b* = 6.8289 (14) Å
*c* = 20.468 (4) Å
β = 97.04 (3)°
*V* = 2679.2 (9) Å^3^
*Z* = 8
Mo *K*α radiation
μ = 0.10 mm^−1^
*T* = 293 K
0.22 × 0.18 × 0.15 mm

#### Data collection

Bruker SMART CCD area-detector diffractometer
Absorption correction: multi-scan (*SADABS*; Sheldrick, 1996 ▶) *T*_min_ = 0.978, *T*_max_ = 0.985
21978 measured reflections
6028 independent reflections
3716 reflections with *I* > 2σ(*I*)
*R*_int_ = 0.049

#### Refinement

*R*[*F* ^2^ > 2σ(*F* ^2^)] = 0.060
*wR*(*F* ^2^) = 0.198
*S* = 1.02
6028 reflections
363 parameters
H-atom parameters constrained
Δρ_max_ = 0.40 e Å^−3^
Δρ_min_ = −0.36 e Å^−3^

### 

Data collection: *SMART* (Bruker, 1997 ▶); cell refinement: *SAINT* (Bruker, 1997 ▶); data reduction: *SAINT*; program(s) used to refine structure: *SHELXS97* (Sheldrick, 2008 ▶); molecular graphics: *SHELXTL* (Sheldrick, 2008 ▶); software used to prepare material for publication: *SHELXTL*.

## Supplementary Material

Crystal structure: contains datablocks global, I. DOI: 10.1107/S160053680902947X/lh2861sup1.cif

Structure factors: contains datablocks I. DOI: 10.1107/S160053680902947X/lh2861Isup2.hkl

Additional supplementary materials:  crystallographic information; 3D view; checkCIF report

## Figures and Tables

**Table 1:** Hydrogen-bond geometry (Å, °)

*D*—H⋯*A*	*D*—H	H⋯*A*	*D*⋯*A*	*D*—H⋯*A*
C4*A*—H4*AA*⋯O4*B*^i^	0.93	2.59	3.408 (3)	147
C1*B*—H1*BA*⋯O4*A*^ii^	0.93	2.50	3.376 (3)	157
C12*B*—H12*B*⋯O4*B*^i^	0.97	2.53	3.420 (3)	152
C13*A*—H13*A*⋯O4*A*^ii^	0.97	2.54	3.405 (3)	149

Symmetry codes: (i) 
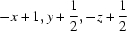
; (ii) 
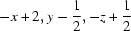
.

**Fig. 1.**
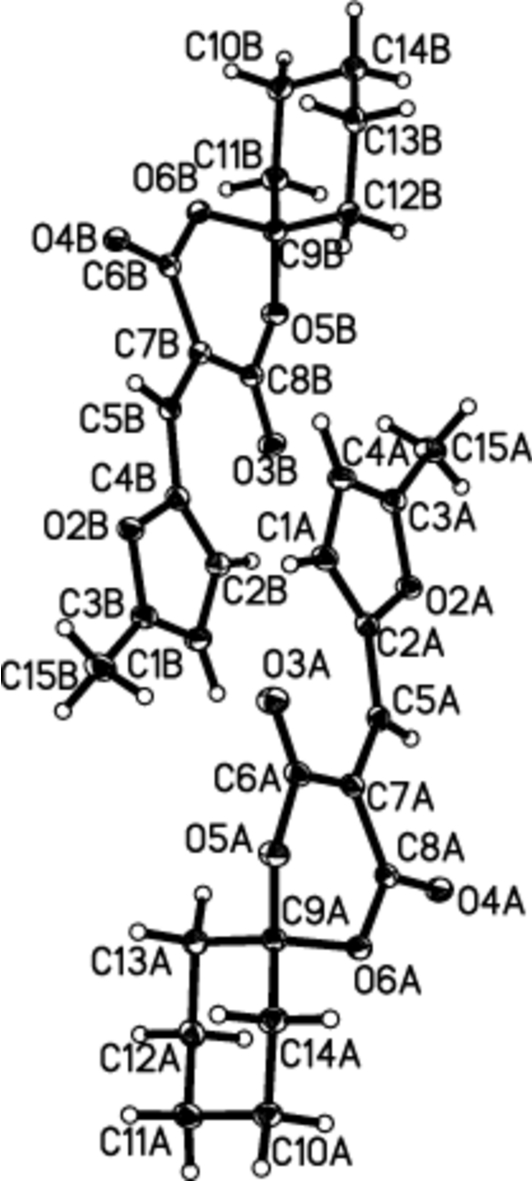
The asymmetric unit of (I), drawn with 30% probability ellipsoids and spheres of arbritrary size for the H atoms.

## supplementary crystallographic information

### Comment

Spiro compounds are widely used in medicine, catalysis and optical material (Lian *et al.*, 2008; Yaozhong *et al.*, 1998; Wei *et al.*, 2008) owing to their interesting conformational features. We report here the synthesis and structure of the title compound, (I), as part of our ongoing studies on new spiro compounds with potentially higher bioactivity and have recently determined the crystal structure of 3-(furan-2-ylmethylene)-1,5-dioxaspiro[5.5]undecane-2,4-dione, (Zeng & Jian, 2009).

The asymmetric unit of (I) is shown in Fig. 1. In both independent molecules, the 1,3-dioxane ring is in an envelope conformation with atoms C9A and C9B forming the flap in each. The mean planes of the other five essentially planar atoms (O5A/O6A/C6A—C8A  and O5B/O6B/C6B—C8B) form dihedral angles of 7.15 (1)° and 6.80 (1)° with the furan ring (O2A/C1A-C4A and O2B/C1B-C4B). The crystal structure is stabilized by weak intermolecular C—H···O hydrogen bonds (Table 1).

### Experimental

A mixture of malonic acid (6.24 g, 0.06 mol) and acetic anhydride(9 ml) in conc. sulfuric acid (0.25 ml) was stirred with water at 303K, After dissolving, cyclohexanone (5.88 g, 0.06 mol) was added dropwise into solution for 1 h. The reaction was allowed to proceed for 4 h. The mixture was cooled and filtered, and then an ethanol solution of 5-methylfuran-2-carbaldehyde (6.60 g,0.06 mol) was added. The solution was then filtered and concentrated. Single crystals were obtained by evaporation of an acetone-petroleum aether (2:1 *v*/*v*) solution of (I) at room temperature over a period of one week.

### Refinement

The H atoms were placed in calculated positions (C—H = 0.93–0.97 Å), and refined as riding with U_iso_(H) = 1.2U_eq_(C) or 1.5U_eq_(methyl C).

### Crystal data

**Table:**

C_15_H_16_O_5_	*F*(000) = 1168
*M**_r_* = 276.28	*D*_x_ = 1.370 Mg m^−^^3^
Monoclinic, *P*2_1_/*c*	Mo *K*α radiation, λ = 0.71073 Å
Hall symbol: -P 2ybc	Cell parameters from 3716 reflections
*a* = 19.314 (4) Å	θ = 3.1–27.5°
*b* = 6.8289 (14) Å	µ = 0.10 mm^−^^1^
*c* = 20.468 (4) Å	*T* = 293 K
β = 97.04 (3)°	Block, yellow
*V* = 2679.2 (9) Å^3^	0.22 × 0.18 × 0.15 mm
*Z* = 8	

### Data collection

**Table:**

Bruker SMART CCD area-detector diffractometer	6028 independent reflections
Radiation source: fine-focus sealed tube	3716 reflections with *I* > 2σ(*I*)
graphite	*R*_int_ = 0.049
φ and ω scans	θ_max_ = 27.5°, θ_min_ = 3.1°
Absorption correction: multi-scan (*SADABS*; Sheldrick, 1996)	*h* = −25→24
*T*_min_ = 0.978, *T*_max_ = 0.985	*k* = −8→8
21978 measured reflections	*l* = −26→26

### Refinement

**Table:**

Refinement on *F*^2^	Primary atom site location: structure-invariant direct methods
Least-squares matrix: full	Secondary atom site location: difference Fourier map
*R*[*F*^2^ > 2σ(*F*^2^)] = 0.060	Hydrogen site location: inferred from neighbouring sites
*wR*(*F*^2^) = 0.198	H-atom parameters constrained
*S* = 1.02	*w* = 1/[σ^2^(*F*_o_^2^) + (0.119*P*)^2^] where *P* = (*F*_o_^2^ + 2*F*_c_^2^)/3
6028 reflections	(Δ/σ)_max_ = 0.001
363 parameters	Δρ_max_ = 0.40 e Å^−^^3^
0 restraints	Δρ_min_ = −0.36 e Å^−^^3^

### Special details

**Table:**

Geometry. All e.s.d.'s (except the e.s.d. in the dihedral angle between two l.s. planes) are estimated using the full covariance matrix. The cell e.s.d.'s are taken into account individually in the estimation of e.s.d.'s in distances, angles and torsion angles; correlations between e.s.d.'s in cell parameters are only used when they are defined by crystal symmetry. An approximate (isotropic) treatment of cell e.s.d.'s is used for estimating e.s.d.'s involving l.s. planes.
Refinement. Refinement of *F*^2^ against ALL reflections. The weighted *R*-factor *wR* and goodness of fit *S* are based on *F*^2^, conventional *R*-factors *R* are based on *F*, with *F* set to zero for negative *F*^2^. The threshold expression of *F*^2^ > σ(*F*^2^) is used only for calculating *R*-factors(gt) *etc*. and is not relevant to the choice of reflections for refinement. *R*-factors based on *F*^2^ are statistically about twice as large as those based on *F*, and *R*- factors based on ALL data will be even larger.

### Fractional atomic coordinates and isotropic or equivalent isotropic displacement parameters (Å^2^)

**Table:**

	*x*	*y*	*z*	*U*_iso_*/*U*_eq_	
O2A	0.77282 (7)	0.9079 (2)	0.18291 (7)	0.0240 (4)	
O3A	0.85666 (7)	1.0679 (3)	0.38985 (7)	0.0302 (4)	
O4A	1.01152 (7)	1.0476 (3)	0.22791 (7)	0.0298 (4)	
O5A	0.97036 (7)	1.1039 (2)	0.41380 (7)	0.0266 (4)	
O6A	1.04784 (7)	1.0940 (2)	0.33323 (7)	0.0250 (4)	
C1A	0.74943 (10)	0.9542 (4)	0.28658 (10)	0.0246 (5)	
H1AA	0.7543	0.9805	0.3315	0.030*	
C2A	0.80226 (10)	0.9507 (3)	0.24675 (9)	0.0226 (5)	
C3A	0.70337 (10)	0.8848 (3)	0.18383 (10)	0.0241 (5)	
C4A	0.68697 (10)	0.9107 (4)	0.24655 (11)	0.0264 (5)	
H4AA	0.6428	0.9012	0.2600	0.032*	
C5A	0.87528 (10)	0.9763 (3)	0.25215 (9)	0.0223 (5)	
H5AA	0.8931	0.9559	0.2125	0.027*	
C6A	0.91304 (10)	1.0628 (3)	0.37011 (10)	0.0234 (5)	
C7A	0.92562 (10)	1.0240 (3)	0.30207 (9)	0.0227 (5)	
C8A	0.99672 (10)	1.0523 (4)	0.28365 (10)	0.0244 (5)	
C9A	1.03783 (10)	1.0380 (3)	0.39939 (9)	0.0225 (5)	
C10A	1.16592 (10)	1.0837 (4)	0.43581 (10)	0.0294 (5)	
H10A	1.1762	1.1199	0.3922	0.035*	
H10B	1.1991	1.1501	0.4677	0.035*	
C11A	1.17412 (10)	0.8647 (4)	0.44459 (10)	0.0295 (5)	
H11A	1.1698	0.8308	0.4899	0.035*	
H11B	1.2203	0.8263	0.4355	0.035*	
C12A	1.11918 (10)	0.7519 (4)	0.39880 (10)	0.0265 (5)	
H12C	1.1237	0.6130	0.4082	0.032*	
H12D	1.1273	0.7724	0.3534	0.032*	
C13A	1.04505 (10)	0.8192 (4)	0.40769 (10)	0.0252 (5)	
H13A	1.0117	0.7541	0.3755	0.030*	
H13B	1.0346	0.7824	0.4512	0.030*	
C14A	1.09154 (10)	1.1511 (4)	0.44459 (10)	0.0275 (5)	
H14C	1.0834	1.1312	0.4899	0.033*	
H14D	1.0868	1.2899	0.4349	0.033*	
C15A	0.66067 (11)	0.8365 (4)	0.12100 (11)	0.0323 (5)	
H15A	0.6659	0.9375	0.0893	0.049*	
H15B	0.6125	0.8269	0.1280	0.049*	
H15C	0.6758	0.7137	0.1049	0.049*	
O2B	0.72619 (7)	0.4699 (2)	0.32748 (7)	0.0260 (4)	
O3B	0.65167 (7)	0.3142 (3)	0.11772 (7)	0.0311 (4)	
O4B	0.48850 (7)	0.3305 (3)	0.27231 (7)	0.0312 (4)	
O5B	0.53852 (7)	0.2773 (3)	0.08899 (7)	0.0289 (4)	
O6B	0.45715 (7)	0.2830 (3)	0.16647 (7)	0.0269 (4)	
C1B	0.81544 (10)	0.4690 (4)	0.26779 (11)	0.0289 (5)	
H1BA	0.8604	0.4789	0.2562	0.035*	
C2B	0.75465 (10)	0.4255 (4)	0.22541 (11)	0.0262 (5)	
H2BA	0.7519	0.3999	0.1805	0.031*	
C3B	0.79632 (10)	0.4941 (4)	0.32922 (11)	0.0269 (5)	
C4B	0.69981 (10)	0.4275 (3)	0.26259 (10)	0.0242 (5)	
C5B	0.62670 (10)	0.4016 (3)	0.25433 (10)	0.0229 (5)	
H5BA	0.6071	0.4197	0.2932	0.027*	
C6B	0.50626 (10)	0.3260 (4)	0.21756 (10)	0.0255 (5)	
C7B	0.57830 (10)	0.3558 (3)	0.20207 (10)	0.0238 (5)	
C8B	0.59395 (10)	0.3186 (4)	0.13503 (10)	0.0249 (5)	
C9B	0.46957 (10)	0.3378 (4)	0.10066 (10)	0.0251 (5)	
C10B	0.34343 (11)	0.2776 (4)	0.06026 (11)	0.0326 (6)	
H10C	0.3122	0.2052	0.0282	0.039*	
H10D	0.3319	0.2447	0.1037	0.039*	
C11B	0.41892 (11)	0.2164 (4)	0.05488 (11)	0.0309 (5)	
H11C	0.4249	0.0789	0.0661	0.037*	
H11D	0.4288	0.2335	0.0099	0.037*	
C12B	0.45988 (10)	0.5554 (4)	0.09071 (10)	0.0263 (5)	
H12A	0.4725	0.5916	0.0479	0.032*	
H12B	0.4905	0.6249	0.1240	0.032*	
C13B	0.38411 (10)	0.6153 (4)	0.09512 (10)	0.0285 (5)	
H13C	0.3738	0.5974	0.1399	0.034*	
H13D	0.3783	0.7530	0.0842	0.034*	
C14B	0.33275 (11)	0.4947 (4)	0.04849 (10)	0.0331 (6)	
H14A	0.2854	0.5296	0.0550	0.040*	
H14B	0.3390	0.5252	0.0033	0.040*	
C15B	0.83547 (11)	0.5452 (4)	0.39357 (12)	0.0354 (6)	
H15D	0.8248	0.4525	0.4262	0.053*	
H15E	0.8225	0.6742	0.4061	0.053*	
H15F	0.8846	0.5421	0.3902	0.053*	

### Atomic displacement parameters (Å^2^)

**Table:**

	*U*^11^	*U*^22^	*U*^33^	*U*^12^	*U*^13^	*U*^23^
O2A	0.0205 (6)	0.0310 (10)	0.0199 (7)	−0.0014 (6)	−0.0002 (6)	−0.0009 (6)
O3A	0.0241 (7)	0.0446 (12)	0.0225 (7)	0.0018 (7)	0.0052 (6)	−0.0025 (7)
O4A	0.0222 (7)	0.0473 (12)	0.0202 (7)	−0.0007 (7)	0.0042 (6)	0.0054 (7)
O5A	0.0221 (7)	0.0372 (11)	0.0205 (7)	0.0017 (6)	0.0022 (6)	−0.0029 (6)
O6A	0.0209 (6)	0.0344 (10)	0.0191 (7)	−0.0023 (6)	0.0007 (6)	0.0042 (6)
C1A	0.0231 (9)	0.0292 (14)	0.0216 (10)	0.0022 (9)	0.0028 (8)	−0.0001 (8)
C2A	0.0232 (9)	0.0244 (13)	0.0195 (9)	0.0016 (8)	−0.0001 (8)	0.0003 (8)
C3A	0.0216 (9)	0.0237 (13)	0.0270 (10)	0.0008 (8)	0.0020 (9)	−0.0017 (8)
C4A	0.0215 (9)	0.0277 (14)	0.0304 (11)	0.0005 (9)	0.0039 (9)	0.0004 (9)
C5A	0.0250 (9)	0.0241 (13)	0.0178 (9)	0.0024 (8)	0.0030 (8)	0.0016 (8)
C6A	0.0215 (9)	0.0287 (14)	0.0195 (9)	0.0008 (8)	0.0003 (8)	−0.0006 (8)
C7A	0.0201 (9)	0.0290 (14)	0.0188 (9)	0.0032 (8)	0.0019 (8)	0.0027 (8)
C8A	0.0223 (9)	0.0298 (14)	0.0203 (10)	−0.0012 (9)	−0.0002 (8)	0.0053 (8)
C9A	0.0202 (9)	0.0284 (13)	0.0190 (9)	0.0002 (8)	0.0032 (8)	0.0018 (8)
C10A	0.0243 (10)	0.0415 (16)	0.0211 (10)	−0.0078 (9)	−0.0018 (9)	0.0002 (9)
C11A	0.0231 (9)	0.0432 (16)	0.0220 (10)	0.0022 (9)	0.0018 (9)	0.0016 (9)
C12A	0.0279 (10)	0.0283 (14)	0.0231 (10)	0.0012 (9)	0.0018 (9)	0.0022 (9)
C13A	0.0242 (9)	0.0326 (14)	0.0185 (9)	−0.0029 (9)	0.0019 (8)	0.0020 (8)
C14A	0.0258 (10)	0.0317 (15)	0.0242 (10)	−0.0063 (9)	−0.0002 (9)	−0.0022 (9)
C15A	0.0273 (10)	0.0378 (16)	0.0300 (11)	−0.0017 (10)	−0.0047 (9)	−0.0052 (10)
O2B	0.0213 (7)	0.0312 (10)	0.0250 (7)	−0.0001 (6)	0.0008 (6)	0.0000 (6)
O3B	0.0247 (7)	0.0426 (12)	0.0272 (8)	0.0018 (7)	0.0076 (6)	−0.0011 (7)
O4B	0.0226 (7)	0.0496 (12)	0.0216 (7)	0.0011 (7)	0.0039 (6)	0.0069 (7)
O5B	0.0224 (7)	0.0407 (11)	0.0238 (7)	0.0009 (7)	0.0031 (6)	−0.0016 (7)
O6B	0.0214 (7)	0.0393 (11)	0.0196 (7)	−0.0029 (6)	0.0010 (6)	0.0053 (6)
C1B	0.0222 (10)	0.0278 (14)	0.0367 (12)	0.0006 (9)	0.0044 (9)	0.0021 (10)
C2B	0.0236 (9)	0.0275 (14)	0.0278 (11)	0.0017 (9)	0.0046 (9)	0.0022 (9)
C3B	0.0220 (9)	0.0237 (13)	0.0341 (11)	0.0007 (8)	0.0001 (9)	0.0000 (9)
C4B	0.0247 (10)	0.0246 (13)	0.0230 (10)	0.0015 (8)	0.0014 (9)	0.0023 (8)
C5B	0.0237 (9)	0.0246 (13)	0.0212 (9)	0.0041 (8)	0.0062 (8)	0.0026 (8)
C6B	0.0231 (9)	0.0293 (14)	0.0234 (10)	0.0007 (9)	0.0002 (9)	0.0049 (9)
C7B	0.0210 (9)	0.0270 (13)	0.0236 (10)	0.0017 (8)	0.0035 (8)	0.0040 (8)
C8B	0.0220 (9)	0.0282 (14)	0.0245 (10)	0.0011 (8)	0.0031 (9)	0.0019 (9)
C9B	0.0215 (9)	0.0338 (14)	0.0199 (9)	−0.0026 (9)	0.0023 (8)	0.0009 (9)
C10B	0.0266 (10)	0.0460 (17)	0.0240 (10)	−0.0102 (10)	−0.0013 (9)	0.0016 (10)
C11B	0.0289 (10)	0.0371 (16)	0.0257 (10)	−0.0044 (10)	−0.0002 (9)	−0.0022 (10)
C12B	0.0265 (10)	0.0335 (14)	0.0191 (9)	−0.0038 (9)	0.0030 (9)	0.0020 (9)
C13B	0.0293 (10)	0.0317 (15)	0.0242 (10)	0.0022 (9)	0.0026 (9)	0.0049 (9)
C14B	0.0256 (10)	0.0517 (18)	0.0209 (10)	−0.0012 (10)	−0.0013 (9)	0.0070 (10)
C15B	0.0294 (11)	0.0390 (17)	0.0352 (12)	−0.0029 (10)	−0.0059 (10)	−0.0007 (11)

### Geometric parameters (Å, °)

**Table:**

O2A—C3A	1.353 (2)	O2B—C3B	1.361 (2)
O2A—C2A	1.391 (2)	O2B—C4B	1.393 (2)
O3A—C6A	1.207 (2)	O3B—C8B	1.211 (2)
O4A—C8A	1.210 (2)	O4B—C6B	1.212 (2)
O5A—C6A	1.364 (2)	O5B—C8B	1.366 (2)
O5A—C9A	1.443 (2)	O5B—C9B	1.442 (2)
O6A—C8A	1.357 (2)	O6B—C6B	1.355 (2)
O6A—C9A	1.443 (2)	O6B—C9B	1.446 (2)
C1A—C2A	1.382 (3)	C1B—C3B	1.364 (3)
C1A—C4A	1.404 (3)	C1B—C2B	1.403 (3)
C1A—H1AA	0.9300	C1B—H1BA	0.9300
C2A—C5A	1.412 (3)	C2B—C4B	1.378 (3)
C3A—C4A	1.371 (3)	C2B—H2BA	0.9300
C3A—C15A	1.478 (3)	C3B—C15B	1.478 (3)
C4A—H4AA	0.9300	C4B—C5B	1.412 (3)
C5A—C7A	1.361 (3)	C5B—C7B	1.368 (3)
C5A—H5AA	0.9300	C5B—H5BA	0.9300
C6A—C7A	1.467 (3)	C6B—C7B	1.478 (3)
C7A—C8A	1.481 (3)	C7B—C8B	1.463 (3)
C9A—C13A	1.508 (3)	C9B—C12B	1.508 (3)
C9A—C14A	1.514 (3)	C9B—C11B	1.516 (3)
C10A—C11A	1.512 (4)	C10B—C14B	1.512 (4)
C10A—C14A	1.540 (3)	C10B—C11B	1.534 (3)
C10A—H10A	0.9700	C10B—H10C	0.9700
C10A—H10B	0.9700	C10B—H10D	0.9700
C11A—C12A	1.534 (3)	C11B—H11C	0.9700
C11A—H11A	0.9700	C11B—H11D	0.9700
C11A—H11B	0.9700	C12B—C13B	1.533 (3)
C12A—C13A	1.536 (3)	C12B—H12A	0.9700
C12A—H12C	0.9700	C12B—H12B	0.9700
C12A—H12D	0.9700	C13B—C14B	1.530 (3)
C13A—H13A	0.9700	C13B—H13C	0.9700
C13A—H13B	0.9700	C13B—H13D	0.9700
C14A—H14C	0.9700	C14B—H14A	0.9700
C14A—H14D	0.9700	C14B—H14B	0.9700
C15A—H15A	0.9600	C15B—H15D	0.9600
C15A—H15B	0.9600	C15B—H15E	0.9600
C15A—H15C	0.9600	C15B—H15F	0.9600
			
C3A—O2A—C2A	107.65 (15)	C3B—O2B—C4B	107.26 (16)
C6A—O5A—C9A	118.84 (15)	C8B—O5B—C9B	119.22 (16)
C8A—O6A—C9A	118.53 (15)	C6B—O6B—C9B	118.84 (15)
C2A—C1A—C4A	107.20 (18)	C3B—C1B—C2B	107.22 (18)
C2A—C1A—H1AA	126.4	C3B—C1B—H1BA	126.4
C4A—C1A—H1AA	126.4	C2B—C1B—H1BA	126.4
C1A—C2A—O2A	108.14 (16)	C4B—C2B—C1B	107.33 (19)
C1A—C2A—C5A	138.96 (19)	C4B—C2B—H2BA	126.3
O2A—C2A—C5A	112.90 (16)	C1B—C2B—H2BA	126.3
O2A—C3A—C4A	109.96 (18)	O2B—C3B—C1B	109.97 (19)
O2A—C3A—C15A	117.54 (17)	O2B—C3B—C15B	116.81 (18)
C4A—C3A—C15A	132.49 (18)	C1B—C3B—C15B	133.19 (19)
C3A—C4A—C1A	107.04 (18)	C2B—C4B—O2B	108.21 (17)
C3A—C4A—H4AA	126.5	C2B—C4B—C5B	139.2 (2)
C1A—C4A—H4AA	126.5	O2B—C4B—C5B	112.55 (17)
C7A—C5A—C2A	134.69 (18)	C7B—C5B—C4B	134.40 (18)
C7A—C5A—H5AA	112.7	C7B—C5B—H5BA	112.8
C2A—C5A—H5AA	112.7	C4B—C5B—H5BA	112.8
O3A—C6A—O5A	117.86 (18)	O4B—C6B—O6B	117.93 (17)
O3A—C6A—C7A	125.73 (18)	O4B—C6B—C7B	125.12 (19)
O5A—C6A—C7A	116.36 (16)	O6B—C6B—C7B	116.91 (17)
C5A—C7A—C6A	124.74 (17)	C5B—C7B—C8B	124.97 (17)
C5A—C7A—C8A	116.08 (18)	C5B—C7B—C6B	115.77 (18)
C6A—C7A—C8A	119.01 (18)	C8B—C7B—C6B	119.03 (18)
O4A—C8A—O6A	118.46 (17)	O3B—C8B—O5B	117.70 (18)
O4A—C8A—C7A	124.77 (19)	O3B—C8B—C7B	125.61 (19)
O6A—C8A—C7A	116.72 (17)	O5B—C8B—C7B	116.63 (16)
O5A—C9A—O6A	109.72 (16)	O5B—C9B—O6B	110.01 (16)
O5A—C9A—C13A	111.06 (16)	O5B—C9B—C12B	111.21 (17)
O6A—C9A—C13A	110.32 (17)	O6B—C9B—C12B	110.41 (17)
O5A—C9A—C14A	106.51 (17)	O5B—C9B—C11B	106.25 (17)
O6A—C9A—C14A	106.11 (16)	O6B—C9B—C11B	105.43 (17)
C13A—C9A—C14A	112.94 (18)	C12B—C9B—C11B	113.30 (18)
C11A—C10A—C14A	111.55 (18)	C14B—C10B—C11B	111.61 (19)
C11A—C10A—H10A	109.3	C14B—C10B—H10C	109.3
C14A—C10A—H10A	109.3	C11B—C10B—H10C	109.3
C11A—C10A—H10B	109.3	C14B—C10B—H10D	109.3
C14A—C10A—H10B	109.3	C11B—C10B—H10D	109.3
H10A—C10A—H10B	108.0	H10C—C10B—H10D	108.0
C10A—C11A—C12A	111.68 (18)	C9B—C11B—C10B	110.70 (19)
C10A—C11A—H11A	109.3	C9B—C11B—H11C	109.5
C12A—C11A—H11A	109.3	C10B—C11B—H11C	109.5
C10A—C11A—H11B	109.3	C9B—C11B—H11D	109.5
C12A—C11A—H11B	109.3	C10B—C11B—H11D	109.5
H11A—C11A—H11B	107.9	H11C—C11B—H11D	108.1
C11A—C12A—C13A	111.25 (18)	C9B—C12B—C13B	111.01 (18)
C11A—C12A—H12C	109.4	C9B—C12B—H12A	109.4
C13A—C12A—H12C	109.4	C13B—C12B—H12A	109.4
C11A—C12A—H12D	109.4	C9B—C12B—H12B	109.4
C13A—C12A—H12D	109.4	C13B—C12B—H12B	109.4
H12C—C12A—H12D	108.0	H12A—C12B—H12B	108.0
C9A—C13A—C12A	110.95 (17)	C14B—C13B—C12B	111.74 (19)
C9A—C13A—H13A	109.4	C14B—C13B—H13C	109.3
C12A—C13A—H13A	109.4	C12B—C13B—H13C	109.3
C9A—C13A—H13B	109.4	C14B—C13B—H13D	109.3
C12A—C13A—H13B	109.4	C12B—C13B—H13D	109.3
H13A—C13A—H13B	108.0	H13C—C13B—H13D	107.9
C9A—C14A—C10A	110.81 (18)	C10B—C14B—C13B	111.28 (18)
C9A—C14A—H14C	109.5	C10B—C14B—H14A	109.4
C10A—C14A—H14C	109.5	C13B—C14B—H14A	109.4
C9A—C14A—H14D	109.5	C10B—C14B—H14B	109.4
C10A—C14A—H14D	109.5	C13B—C14B—H14B	109.4
H14C—C14A—H14D	108.1	H14A—C14B—H14B	108.0
C3A—C15A—H15A	109.5	C3B—C15B—H15D	109.5
C3A—C15A—H15B	109.5	C3B—C15B—H15E	109.5
H15A—C15A—H15B	109.5	H15D—C15B—H15E	109.5
C3A—C15A—H15C	109.5	C3B—C15B—H15F	109.5
H15A—C15A—H15C	109.5	H15D—C15B—H15F	109.5
H15B—C15A—H15C	109.5	H15E—C15B—H15F	109.5

### Hydrogen-bond geometry (Å, °)

**Table:**

*D*—H···*A*	*D*—H	H···*A*	*D*···*A*	*D*—H···*A*
C1A—H1AA···O3A	0.93	2.26	2.878 (3)	123
C4A—H4AA···O4B^i^	0.93	2.59	3.408 (3)	147
C1B—H1BA···O4A^ii^	0.93	2.50	3.376 (3)	157
C2B—H2BA···O3B	0.93	2.26	2.884 (3)	124
C12B—H12B···O4B^i^	0.97	2.53	3.420 (3)	152
C13A—H13A···O4A^ii^	0.97	2.54	3.405 (3)	149

Symmetry codes: (i) −*x*+1, *y*+1/2, −*z*+1/2; (ii) −*x*+2, *y*−1/2, −*z*+1/2.
